# The detection of hTERC amplification using fluorescence in situ hybridization in the diagnosis and prognosis of cervical intraepithelial neoplasia: a case control study

**DOI:** 10.1186/1477-7819-10-168

**Published:** 2012-08-21

**Authors:** Geping Yin, Juan Li, Tongyu Zhu, Xiaoli Zhao

**Affiliations:** 1 Department of Obstetrics & Gynecology, Jinan Military General Hospital, 25 Shifan Road, Jinan, 250031, China

**Keywords:** Fluorescence in situ hybridization, Cervical intraepithelial neoplasia, Cervical carcinoma, Telomerase

## Abstract

**Background:**

Currently the routine non-invasive screening methods for cervical intraepithelial neoplasia (CIN) and cervical cancer are Thinprep cytology test (TCT) and human papillomavirus testing. However, both methods are limited by the high false positive and false negative rates and lack of association with patients’ prognosis, especially for the early detection of pro-malignant CIN. The aim of the study was to investigate the role of genomic amplification of human telomerase gene (hTERC) in the diagnosis and prognosis of CIN.

**Methods:**

The study group consisted of specimens of exfoliated cervical cells from 151 patients, including 27 with CIN I, 54 with CIN II/III, 17 with carcinoma in situ, and 28 with invasive squamous carcinoma, as well as 25 patients who were at 2-year follow-up after either Loop Electrosurgical Excision treatment (n = 11) or radical surgery (n = 14). hTERC amplification was detected by dual-color interphase fluorescence in situ hybridization (FISH), and the results were compared with TCT and histologic examination. The final diagnosis was determined by the pathological examination. The control group consisted of specimens of exfoliated cervical cells from 40 normal women.

**Results:**

The percentage of cervical exfoliated cells with positive hTERC amplification and incidence rates of hTERC amplification were 9.2% ± 4.6% and 44.4% (12/27) respectively in patients with CIN I; 16.0% ± 14.4% and 85.1% (46/54) in patients with CIN II/III; 19.7% ± 13.3% and 88.3% (15 /17) in patients with carcinoma in situ; 47.0% ± 25.2% and 100% (28/28)in patients with invasive squamous carcinoma. There was statistically significant difference between the control and study group (P <0.01), and between the patients with various diseases within the study group (P <0.05).

**Conclusion:**

The detection of genomic amplification of hTERC using FISH is a non-invasive and effective approach for CIN.

## Background

Studies have shown that the incidence of cervical cancer is closely associated with human papillomavirus (HPV) infection, with HPV16 and HPV18 being the most common subtypes [1-3]. Currently routine non-invasive screening methods for cervical intraepithelial neoplasia (CIN) and cervical cancer are Thinprep cytology test (TCT) and HPV testing. However, both methods are limited by the high false positive and false negative rates and lack of association with patients’ prognoses [4]. Therefore, exploring new non-invasive tests to assist the diagnosis of cervical lesions, especially the early detection of pro-malignant CIN, is important in improving the diagnosis and treatment of cervical cancer [5].

In recent years, studies have found that the overexpression of *human chromosome telomerase* gene (*hTERC*) is a biological marker for cervical cancer [6]. Fluorescence *in situ* hybridization (FISH) can be used to study the chromosome of cervical cells or examine intra-cellular genetic information within cells, and has been applied in the diagnosis of cervical cancer [7,8]. Currently the main treatment for CIN is to retain the uterus, such as the loop electrical excision procedure (LEEP) [9], but the risk of canceration still exits after treatment, especially for patients with CIN II to III who were previously infected with HPV, and long-term follow-up is usually necessary [10-14]. In this study, FISH was used to detect *hTERC* amplification before surgery in cervical exfoliated cells of patients with CIN, and the results were compared with normal cervical epithelium and exfoliated cells of patients with cervical cancer. A retrospective analysis of patients with CIN who reached 24-month post-operative follow-up was also performed to assess the value of using *hTERC* amplification in the clinical diagnosis and prognosis of CIN. To the best of our knowledge, this is the first study on this interesting issue.

## Methods

### Study population

Inclusion criteria: cervical cell specimens prior to treatment were collected from patients who visited the gynecology clinic at Jinan Military General Hospital from December 2007 to December 2009. All patients undertook cytological examination, colposcopy-directed cervical biopsy [15,16], and received the LEEP or surgery. The study sample comprised exfoliated cervical cell specimens from 151 patients (average age 43.5 ± 8.5 years. Mean ± SD), including 81 cases of CIN (27 grade I, 54 grade II to III, average age 38.5 ± 2.7 years) and 45 cases of cervical squamous cell carcinoma (17 carcinoma *in situ*, 28 invasive carcinoma, average age 47.3 ± 13.5 years). There were also 25 cervical cell smears collected from patients who were followed for two years after treatment, including 11 patients with CIN III treated by the LEEP (average age 39.0 ± 4.5 years), and 14 patients with squamous cervical carcinoma who received radical hysterectomy (average age 48.5 ± 10.5 years). All patients provided informed consent to the use of their cervical cell specimens in this study. This research was also approved by the institutional review board prior to initiation. The control group included exfoliated cervical cell specimens from 40 women who were healthy and normal from routine gynecologic examination (negative for intraepithelial lesion or malignancy (NILM) from TCT, average age 41.7 ± 8.1 years). All cases were confirmed by pathological examination.

### Methods

For the collection of cervical cell specimens, a TCT specimen brush was inserted 2 to 3 cm into the cervical canal, and rotated 5 times around the axis of the canal. The cervical brush was kept in the TCT preservation solution. Slides were made using the TCT smearing slide machine, and Papanicolaou-stained. The remaining cells were stored at 4 K for *hTERC* amplification detection using FISH. The slides were processed twice (5 minutes each time) with a 2-fold dilution of sodium citrate buffer (SSC), with 0.1 mol/L hydrochloric acid solution for 10 minutes, with pepsin hydrochloric acid solution at 37 K for 8 minutes, 2-fold dilution of SSC twice more (5 minutes each), 70%, 85%, 100% gradient ethanol solution at room temperature for 3 minutes each, and then heated at 56 K for 3 minutes.

Dual color site-specific chromosome /centromeric probes (hTERC/CSP3 DNA) were provided by GP Medical Technologies Inc. (Beijing, China). CSP3 was used as control probe. The hybridization of the hTERC DNA probe with the chromosome 3 3q 26.3 would show a red fluorescence signal and the hybridization of CSP3 DNA with the centromere of chromosome 3 (3p11.1-q11.1) would show a green fluorescence signal. For genetic variability and hybridization, 70% formamide/2 × SSC denaturation solution was pre-heated in warm water (73 K), and the probing mixture was then added for 5 minutes, followed by 70%, 85%, 100% ethanol gradient at −20 K. for 3 minutes each. Then 10 μL denatured probing mixture was added to the hybridization area of the slide, which was then covered and embedded in mounting medium, incubated at 42 K overnight. On the next day, the slides were placed in three bottles of formamide washing solution at 46 K for 10 minutes each. The coverslip was removed in the first bottle, and slides were washed in the second and third bottle. Then the slides were placed in 2 × SSC (B solution) at 46 K for 10 minutes, air dried in the dark, then read under a fluorescent microscope after staining.

A threshold for positive cervical intraepithelial hTERC amplification using FISH was established. The interphase cell fluorescence hybridization signal was observed under three-color fluorescence microscopy. Image analysis was performed using fluorescence *in situ* hybridization analysis software (GP Medical Technologies Inc.) to evaluate the hybridization of *hTERC* and *CSP3* on the smear. For the control group, based on the results of FISH test, the percentage of cells with positive *hTERC* amplification was defined as the percentage of cells with more than two red fluorescent signals. The mean and SD was then calculated for the control group according to the following formula:

For the study group, a positive test was defined as the percentage of cells with more than two red signals higher than the positive threshold, indicating abnormal *hTERC* amplification. If the percentage was lower than the positive threshold, then the test was negative, indicating normal *hTERC* amplification. If the percentage was equal to the positive threshold, the result was reassessed after increasing the number of cells examined. The incidence rate of *hTERC* amplification was the percentage of positive *hTERC* amplification cases in each group.

For statistical analysis, the row × column chi square test (R × C *Χ*^*2*^) test (SPSS, Version 17) was used to compare the positive rates between groups and the Wilcoxon signed ranks test was used to compare continuous variables between groups.

## Results

### The association between the results of cytological test (TCT) and pathological diagnosis

In the 40 control cases, TCT showed 3 cases of atypical squamous epithelium of undetermined significance (ASCUS), later shown on pathologic examination to be inflammation. There was no low-grade squamous intraepithelial lesion (LSIL), high-grade squamous intraepithelial lesion (HSIL) or squamous cell carcinoma of the cervix (SCC) (Table 1).

**Table 1:** TCT diagnosis of cervical epithelial cells in control and study groups

**Pathologic diagnosis**	**Number**	**NILM**	**ASCUS**	**LSIL**	**HSIL**	**SCC**
Control group	40	37 (92.5)	3 (7.5)	0 (0.0)	0 (0.0)	0 (0.0)
Study group	151					
CIN I	27	2 (7.4)	7 (25.9)	11 (40.8)	7 (25.9)	0 (0.0)
CIN II/III	54	0 (0.0)	7 (12.9)	21 (38.9)	26 (48.2)	0 (0.0)
Carcinoma in situ	17	0 (0.0)	4 (23.5)	2 (11.8)	11 (64.7)	0 (0.0)
Invasive Carcinoma	28	0 (0.0)	0 (0.0)	0 (0.0)	0 (0.0)	28 (100.0)
CIN LEEP post-treatment	11	7 (63.6)	4 (36.4)	0 (0.0)	0 (0.0)	0 (0.0)
Cervical cancer post-treatment	14	7 (50.0)	7 (50.0)	0 (0.0)	0 (0.0)	0 (0.0)

Results presented as number (%). TCT, thinprep cytologic test; Number, number of cases; NILM, negative for intraepithelial lesion or malignancy; ASCUS, atypical squamous cells of undetermined significance; LSIL, low-grade squamous intraepithelial lesion; HSIL, high-grade squamous intraepithelial lesion; SCC, squamous cell carcinoma; CIN, cervical intraepithelial neoplasia; LEEP, loop electrical excision procedure.

### hTERC Amplification in control group and determination of positive threshold

Among the normal cervical epithelial cells, there were two red and two green signals in nucleus during cell interphase. In the control group, on average 2.4% of specimens showed more than two red fluorescence signals (*hTERC* amplification) on the whole slide (SD 1.2%), and accordingly the positive threshold was set to 6.0%. The incidence rate of positive *hTERC* amplification in the control group was 0.0%.

### The relationship between hTERC amplification in exfoliated cervical epithelial cells and the pathological diagnosis in the study group

**Figure 1:**
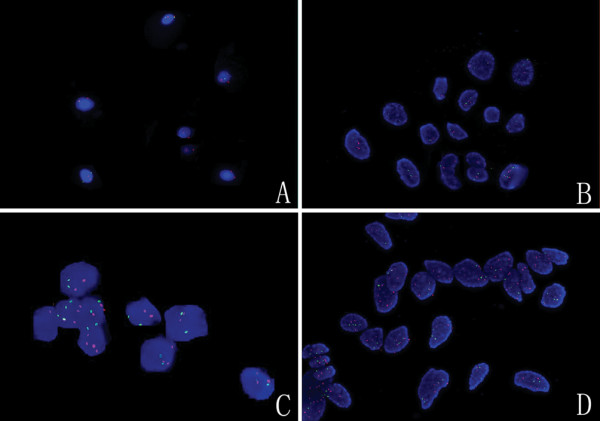
**Photographs of *hTERC *amplification using FISH in the normal cervix, CIN I, CIN III and cervical carcinoma.** (**A)** Normal cervix. Among the normal cervical epithelial cells, there were two red and two green signals in nuclei during cell interphase. (**B**) CIN I. The *hTERC* amplification in heterogeneous cells was demonstrated as more than two red signals and no less than two green signals in the nucleus during cell interphase. The *hTERC* amplification test showed that the percentage of cells with more than two red signals was on average 9.2%. (**C**) CIN III. The hTERC amplification test showed that the percentage of cells with more than two red signals was on average 16.0%. (**D**) Cervical cacinoma. The *hTERC* amplification test showed that the percentage of cells with more than two red signals was on average 47.0%. *hTERC*, human telomerase mRNA component gene; FISH, fluorescence *in situ* hybridization; CIN, cervical intraepithelial neoplasia.

*hTERC a*mplification in heterogeneous cells demonstrated more than two red signals and no less than two green signals in the nucleus during cell interphase. For the 81 CIN cases, the *hTERC* amplification test showed that the percentage of cells with more than two red signals was 13.5% (SD 12.4%), the incidence of positive case was 71.6% (58/81), the sensitivity was 71.6% and the specificity was 100%. As summarized in Table 2, the percentage of *hTERC* amplification positive cells and the incidence of positive case were 9.2% (SD 4.6%) and 44.4% (12/27) for CIN I cases (the sensitivity was 44.4%), 16.0% (SD 14.4%), 85.1% (46/54) for CIN II to III cases (the sensitivity was 85.1%) 19.7% (SD 13.3%), 88.3% (15 /17) for carcinoma *in situ* cases, 47.0% (SD 25.2%) and 100% (28/28) for invasive carcinoma cases (the sensitivity was 100%) respectively. The specificity was 100% for all these subgroups (Figure 1).

**Table 2:** ***hTERC *amplification measured by FISH in exfoliated cervical epithelial cells**

**Diagnosis**	**Number**	**Percentage of positive cell (mean ± SD)**	**Incidence of positive cases (number positive/total number)**
Control group	40	2.4 ± 1.2	0 (0.0)
Study group	151		
CIN I	27	9.2 ± 4.6*	44.4% (12 /27)*
CIN II/III	54	16.0 ± 14.4*	85.1% (46/54)*
Carcinoma *in situ*	17	19.7 ± 13.3*	88.3% (15 /17)*
Invasive Carcinoma	28	47.0 ± 25.2 *	100% (28 /28)*
CIN LEEP post-treatment	11	7.2 ± 2.7*	18.2% (2 /11)
Cervical cancer post-treatment	14	7.1 ± 2.0*	14.3% (2 /14)

FISH, fluorescence *in situ* hybridization; *hTERC*, human telomerase mRNA component gene; Number, number of cases; CIN, cervical intraepithelial neoplasia; LEEP, loop electrical excision procedure. *P < 0.05 (compared with the control group).

With the increase of pathological grade of the cervical lesion, the percentage of exfoliated cells with more than two red signals in the *hTER*C amplification test was also elevated in the study group (except the cases with 2-year post-treatment follow-up). The percentages of positive cell in patients with CIN, carcinoma *in situ* and invasive carcinoma were all statistically significantly higher than in the control group (Table 2).

### The relationship between hTERC amplification and TCT among exfoliated cervical epithelial cells in the study group

The rates of positive *hTERC* amplification among 4 types of abnormal cervical epithelial cells (ASCUS, LSIL, HSIL and SCC) were all significantly higher than among normal epithelial cells in the control group (*P* < 0.05). There was no significant difference in the rate of positive *hTERC* amplification between each type of abnormal cells within the study group (*P* > 0.05). The percentages of cells with positive *hTERC* amplification differed between various types of abnormal cells (*P* < 0.05), and tended to be higher from ASCUS to SCC (Table 3).

**Table 3:** **Relationship between TCT diagnosis and *hTERC *amplification in exfoliated cervical epithelial cells**

**TCT Diagnosis**	**Number**	**Percentage of positive cells (mean ± SD)**	**Incidence of positive cases (%)**
Control group	40	2.4 ± 1.2	0 (0.0)
Study group	151		
NILM	16	3.2 ± 2.1	0 (0.0)
ASCUS	29	8.7 ± 6.3*	13 (44.8)*
LSIL	34	10.8 ± 4.8*	23 (67.6)*
HSIL	44	23.9 ± 15.0*	41 (93.2)*
SCC	28	31.07 ± 22.0*	28 (100)*

TCT, thinprep cytologic test; FISH, fluorescence *in situ* hybridization; *hTER*C, human telomerase mRNA component gene; Number, number of cases; NILM, negative for intraepithelial lesion or malignancy; ASCUS, atypical squamous cells of undetermined significance; LSIL, low-grade squamous intraepithelial lesion; HSIL, high-grade squamous intraepithelial lesion; SCC, squamous cell carcinoma. *P <0.05(compared to the control group).

### Comparison of sensitivity between TCT and hTERC amplification in the detection of cervical intraepithelial lesions by FISH using pathological diagnosis as reference

In the study group, out of 27 CIN-I cases confirmed by pathological examination, TCT detected 40.8% of LSIM (11/27). Also, TCT identified 48.2% (26/54) of HSIL in CIN II to III cases, 64.7% (11/17) of HSIL in cases with carcinoma *in situ*, and 100% (28/28) of SCC in cases with cervical carcinoma. In contrast, the positive rate of *hTER*C was 44.4% (12 /27) for CIN-I, 85.1% (46/54) for CIN II to III, 88.3% (15/17) for carcinoma *in situ* and 100% (28/28) for cervical invasive carcinoma. The *Χ*^2^ test showed that FISH had higher sensitivity than TCT in detecting all types of high-grade cervical intraepithelial lesions (*P* < 0.05), except cervical invasive carcinoma. For the 15 cases with 2-year post-treatment follow-up, the results of TCT were NILM and ASCUS, consistent with the negative findings of *hTERC* amplification.

### hTERC amplification in exfoliated cervical cells at pre- and post-operation among patients with normal 2-year follow-up

The comparison of *hTERC* amplification in exfoliated cervical or vaginal caecum cells at pre-operation and 2-year post-operation in 25 patients, including 11 cases of CIN III and 14 cases of cervical invasive carcinoma, with normal follow-up, showed that after LEEP or radical hysterectomy (using pathological diagnosis as the reference), both the percentage of *hTERC* amplification-positive cells and rate of positive cases decreased significantly (*P* < 0.05) (Table 4), indicating that LEEP and radical surgery were able to significantly reduce the number of cells with positive *hTERC* amplification.

**Table 4:** The comparison of hTERC amplification at pre- and post-operation among patients with normal 2-year follow-up

**Pathologic Diagnosis**	**N**	**Percentage of positive cells (± SD%)**	**Incidence of positive cases (number incident cases/total)**		
		**Pre-operation**	**Post-operation**	**Pre-operation**	**Post-operation**
CIN LEEP	11	11.2 ± 4.1	7.2 ± 2.7*	72.7% (8/11)	18.2%* (2/11)*
Radical hysterectomy	14	45.7 ± 21.6	7.1 ± 2.0*	100% (14/14)	14.3%* (2 /14)

*hTERC*, human telomerase mRNA component gene; CIN, cervical intraepithelial neoplasia; LEEP, loop electrical excision procedure. *P < 0.05 (compared with pre-operation).

## Discussion

HPV infection is a major risk factor for cervical cancer [1,2,4]. About 95% of patients with CIN carry HPV oncogenes, but only a few cases eventually develop invasive cervical cancer. Therefore, HPV infection is not the only factor for malignant transformation. Other factors may also contribute to the development of cervical cancer [5]. Studies have found that during the transforming process from atypical anomalies to cancer, almost all cervical epithelial cells showed abnormal *hTERC* amplification [6,8,17,18]. Human chromosome telomerase comprises telomerase mRNA (hTERC), telomerase reverse transcriptase (hTERT) and telomerase-binding protein (hTP1). The mutation of *hTERC* could lead to functional change of telomerase, which in turn causes chromosomal abnormalities [18,19]. Therefore, the detection of *hTERC* amplification could be regarded as a marker of high grade lesion in future. After 1 to 3 years follow-up, patients with CIN I/II and positive *hTERC* amplification were more likely to progress to CIN III than those with negative *hTERC* amplification, indicating that specific gene mutation is the key for the development of CIN into invasive carcinoma [8]. In fact, the fundamental differences between ASCUS, reversible mild CIN and progressive CIN are the genetic characteristics. From CIN II/III to cervical squamous cell carcinoma, the copy number of *hTERC* also steadily increased [19].

This study showed that positive *hTERC* amplification is not only an important biological marker for cervical cancer [20-22], but also an important indicator of pre-malignant CIN.

In the 40 control subjects, TCT showed 3 cases of ASCUS, which were later confirmed on pathologic examination to be inflammation, and there were no LSIL, HSIL or SCC cases. For the 81 CIN cases in the study group, the *hTERC* amplification test showed that 13.5% (SD 12.4%) of cells were positive (two red signals) , and the incidence rate of positive case was 71.6% (58/81). Of them, the percentage of positive cells and the incidence of positive cases were 9.2% (SD 4.6%) and 44.4% (12/27) for CIN I cases respectively, and 16.0% (SD 14.4%), 85.1% (46/54) for CIN II to III cases respectively. This suggested that the higher the pathological grade of cervical lesion, the higher the likelihood of being detected by *hTERC* amplification test in exfoliated cervical cells. In this study, the rate of positive *hTERC* amplification was 100% in patients with invasive cervical cancer. Patients with CIN, carcinoma *in situ* and invasive carcinoma also had a higher percentage of positive cells than control patients, and the differences were statistically significant (*P* < 0.05). In addition, the percentage of positive cells differed by pathological grade, and gradually increased with increase in pathological grade within the study group.

Therefore, this method is important for CIN and cervical cancer screening, especially for identifying pre-malignant CIN. CIN with positive *hTERC* amplification, regardless of the pathological grade, should be given special attention in the clinical management.It should be noted that in this study the comparison of *hTERC* amplification results in the local exfoliated cells at pre-operation and 2-year post-operation showed that after LEEP or radical hysterectomy, both the percentage of positive cells and incidence of positive cases were significantly reduced, indicating that LEEP and radical surgery might be able to significantly reduce the number of cells with *hTERC* amplification.

The significance of establishing criteria to determine hTERC amplification

As for the issue of establishing positive criteria for *hTERC* amplification, previous research defined that if the percentage of cells with more than two red signals in nuclei during interphase reached 2.3%, the result was positive [8]. This criterion improved the detection rate of CIN, although it cannot distinguish between LSIL and HSIL, and also increased the false positive rate.

## Conclusion

The detection of genomic amplification of *hTERC* using FISH is a non-invasive and effective approach for CIN. It is not only an important biological marker for cervical cancer, but also an important indicator of pre-malignant CIN.

### Study limitation

This study was conducted in a single hospital, limited by small number of cases and short duration of follow-up. Further research is warranted to investigate other relevant issues, for example whether positive *hTERC* amplification at 2-year follow-up after LEEP treatment among CIN patients indicates the recurrence of CIN or progression to cervical cancer.

## Abbreviations

ASCUS: atypical squamous epithelium of undetermined significance; CIN: cervical intraepithelial neoplasia; FISH: fluorescence *in situ* hybridization; HPV: human papillomavirus; HSIL: high-grade squamous intraepithelial lesion; hTERC: human telomerase gene; hTERT: telomerase reverse transcriptase; hTP1: telomerase-binding protein; LEEP: loop electrical excision procedure; LSIL: low-grade squamous intraepithelial lesion; NILM: negative for intraepithelial lesion or malignancy; SCC: squamous cell carcinoma; SSC: sodium citrate buffer; TCT: thinprep cytology test.

## Competing interests

The authors declare that the authors have no conflicts of interest.

## Authors’ contributions

GP conceived and designed the experiments, performed the experiments and wrote the paper. JL performed the experiments. TY analyzed the intellectual data; XL contributed materials and analysis tools. All authors read and approved the final manuscript.
